# Hemiataxia: A Novel Presentation of Anti-NMDA Receptor Antibody Mediated Encephalitis in an Adolescent

**DOI:** 10.1155/2017/1310465

**Published:** 2017-12-03

**Authors:** Greg D. Phillips, Gillian N. Jones, Maureen Callaghan, Marc P. DiFazio

**Affiliations:** ^1^Department of Neurology, Madigan Army Medical Center, Joint Base Madigan McChord, 9040 Jackson Ave, Tacoma, WA 98431, USA; ^2^Department of Neurology, Children's National Health System, 111 Michigan Ave NW, Washington, DC 20010, USA

## Abstract

Anti-NMDA receptor antibody associated encephalitis as a cause of new-onset neuropsychiatric manifestations in children and adults can represent a significant diagnostic challenge for clinicians. Clinical signs often include encephalopathy, new-onset psychosis, and movement phenomenon. Although orofacial dyskinesias were initially identified as a characteristic movement phenomenon in this type of encephalitis, an expanded range of abnormalities has recently been reported, including isolated ataxia. We report a case of isolated hemiataxia in a young adult with mild initial psychiatric manifestations. A personal and family history of preceding neuropsychiatric symptoms produced diagnostic confusion and resulted in a significant diagnostic and therapeutic delay. Our case confirms the unilateral movement manifestations that have been emphasized in recent reports. Additionally, it confirms the need for involvement of neurologic as well as psychiatric services in the evaluation of such cases and emphasizes the importance of the neurologic examination in presentations with an initial psychiatric predominance.

## 1. Introduction

Anti-NMDA receptor antibody associated encephalitis is an increasingly recognized cause of new-onset neuropsychiatric manifestations in children and adults [1]. Clinical signs often include encephalopathy, new-onset psychosis, and movement phenomenon [2]. Although orofacial dyskinesias were initially identified as a characteristic movement phenomenon in this type of encephalitis, an expanded range of abnormalities has recently been reported, including isolated ataxia [3, 4]. We report a case of isolated hemiataxia in a young adult with mild initial psychiatric manifestations. A personal and family history of preceding neuropsychiatric symptoms produced diagnostic confusion and resulted in a significant diagnostic and therapeutic delay. Our case appears to confirm the unilateral movement manifestations that have been emphasized in recent reports [5]. Additionally, it emphasizes the need for involvement of neurologic as well as psychiatric services in the evaluation of such cases and demonstrates the importance of the neurologic examination in presentations with an initial psychiatric predominance.

## 2. Case Presentation

Our patient, an 18-year-old female, was referred to neurology with a diagnosis of schizoaffective disorder after a possible first-time seizure. She had experienced previous mood lability and attentional impairments as a teenager and had been treated for both during that time. However, she had been off medications for 5 years and had been doing well in the recent past. Grades and athletic functioning were above average, and she played three sports in high school and was in mainstream classes. Her family history was notable for a history of maternal depression, psychogenic nonepileptic seizures, and anxiety, as well as a maternal grandmother with a reported history of schizophrenia and bipolar disorder. Eight months prior to the patient's referral to neurology, she experienced a self-limited illness characterized by diarrhea, headache, and vomiting. This was managed at home, although she was seen by her primary care provider and diagnosed with a viral gastroenteritis. Approximately one month later, she began to experience insomnia, mood lability, and weight loss. Memory issues ensued, and the parents reported that she began to frequently lose objects and had difficulty remembering bus routes. Personal hygiene suffered, and after 4 months of worsening symptoms, she began to experience weekly episodes of urinary incontinence. There were no other autonomic manifestations. Throughout the course of the subacute illness, she experienced a “tremor” of the left upper extremity when attempting to manipulate or reach for objects, as well as dragging her left foot when ambulating. She received ongoing psychiatric treatment and monitoring, and her movement phenomenon was attributed to a psychogenic cause, and a diagnosis of likely schizoaffective disorder was made. After 7 months of symptoms, she experienced a spell, characterized by clonic movements of the left hand, followed by tonic stiffening of her entire body, with right sided head and neck deviation, lasting 30 seconds, followed by confusion and sedation. She recovered to baseline after 30 minutes. After this episode, she was referred to neurology. On initial presentation to our service, her spell was attributed to a first-time seizure by history. Mental status examination demonstrated a distracted and labile demeanor. Cognition was impaired on both bedside and formal neuropsychological testing with impairments in memory, attention, and reasoning. Neurologic examination demonstrated left sided dysmetria as well as gait disturbance arising from left lower extremity coordination impairment. MRI of the brain revealed increased FLAIR signal of the left hippocampus. No cerebellar or contralateral brain findings were identified as potential substrate for her unilateral ataxia. Electroencephalogram was normal. The combination of her ataxia, cognitive decline, and incontinence prompted an extensive laboratory evaluation for systemic conditions as well as inborn errors of metabolism. Spinal tap was performed, with testing for autoimmune and inflammatory conditions. Testing was positive only for the presence of NMDA receptor antibodies in the spinal fluid (NMDA-R Ab IF Titer Assay, CSF Positive 1 : 4 and reference < 1 : 2, Mayo Clinic Laboratories) with no evidence of active infection or inflammation. Extensive evaluation for a neoplastic substrate, specifically ovarian teratoma, was negative. Because of the extended course experienced by the patient she was started on an aggressive regimen of immune modulation, including concurrent intravenous immunoglobulin, IV methylprednisolone, and rituximab. Although her parents report that psychiatric symptoms were improving after 3 months of treatment, she continues to require medical treatment for her psychiatric manifestations and has been unable to return to school or employment. Additionally, her movement abnormalities persist.

## 3. Discussion

Anti-NMDA receptor antibody encephalitis is increasingly identified as an etiology for noninfectious encephalitis in children and adults [6]. A classic presentation of the condition would be acute onset psychosis/encephalopathy with associated orofacial dyskinesias, without clear precipitant or injury, leading to progressive decline and coma in some [7]. Tumor as a substrate for a paraneoplastic etiology is not uncommon [8]. As awareness of the condition has grown, a broadening of the phenotype has become evident, associated in some with seizures and gait disturbances [9, 10]. Importantly, a growing number of movement disorders have been identified as well [4, 10–12]. Our patient's initial presentation with new-onset “tremor” in association with psychiatric decline is characteristic in that there was a clear association with movement phenomenon and neurobehavioral deterioration. However, to our knowledge, a unilateral presentation of ataxia has not been described to date. Movement phenomena in children and adults with psychiatric conditions are not uncommon and include tic, stereotypy, chorea, and drug induced dyskinesias [13, 14]. Depending on the etiologic substrate, these manifestations may be of variable degree and sometimes subtle in their presentation, with psychiatric signs outweighing manifestations of movement impairment [15, 16]. A detailed neurologic examination is therefore indicated in all individuals with unexplained neuropsychiatric presentations, especially if clinical signs indicate neurologic dysfunction beyond isolated psychiatric impairments [17]. Our patient's manifestations of ataxia were initially ascribed to psychogenic tremor, and coordination disturbances can appear to be semirhythmic in character (as with tremor) as the patient attempts to reach a target in the environment. However, a variety of means can be used to identify the clear disturbance in amplitude modulation seen in ataxia/dysmetria, such as repetitive finger to object testing or testing of the ability to rhythmically and quickly modify hand position (finger, hand tapping, etc.). Ataxia is a common, nonspecific accompaniment of a number of congenital, posttraumatic, metabolic, and autoimmune conditions [18]. Manifestations can be bilateral or unilateral and variably affect the appendicular or axial musculature. Signs are typically related to a dysfunction of amplitude of movement and coordination, often leading to gait disturbance and impairments of fine motor functioning. Although typically localized to the cerebellum, ataxia may occur resulting from injury to a variety of neuroanatomic regions. Because it may be semirhythmic as the patient attempts to correct the movement trajectory, it can be confused with tremor. The coarse and variable amplitude in some may also be reminiscent of a psychogenic manifestation [19]. Formal neurologic examination at 8 months of symptoms demonstrated, however, a clear isolated dysmetria affecting the left upper extremity and mild left sided ataxia. In our case, the abnormal movements were initially attributed to a psychogenic etiology. Although psychogenic movement disorders are common, the manifestations of this patient upon formal neurologic evaluation were clearly attributable to a neurologic etiology. The combination of a unilateral movement abnormality in association with the psychiatric manifestations placed her case outside the realm of an isolated psychiatric condition and warranted further diagnostic evaluation. Our case emphasizes both the expanded spectrum of movement phenomenon that might be seen in cases of anti-NMDA receptor antibody encephalitis and the variability of the encephalopathy at presentation and throughout the course. Additionally, recent reports indicate a propensity for unilateral presentations of the movement phenomenon as was clearly demonstrated in our patient with more than 8 months of hemiataxia without contralateral findings [5]. Testing for anti-NMDA antibody disorders has become readily available, with serum and spinal fluid analysis typically demonstrating positivity in affected individuals. The presence of the antibodies themselves is considered pathologic, and the autoimmune dysregulation that underlies this condition appears to be triggered by a number of precipitants, including paraneoplastic and infectious entities [20]. Treatment for the autoimmune disorder is most effective when initiated early in the course, and, unfortunately, our patient was not diagnosed until approximately 8 months after disease onset. This may portend a more challenging prognosis, and a possible increased rate of disease recurrence and permanent neuropsychiatric deficits were discussed with the family as a result [21]. Additionally, screening for a neoplastic substrate is important as early recognition and treatment of an underlying tumor may improve neurologic outcome. Treatment of these conditions typically involves a combination of immunosuppressive and immunomodulating agents in hopes of decreasing abnormal antibody production and eliminating the harmful effects of antibodies already circulating [22]. Improvements are typically seen within days to weeks. However, a prolonged course refractory to medical therapy is possible, and multiple agents for immune suppression may be necessary. At present, formal recommendations regarding duration of therapy are unknown [1]. A significant number of patients relapse, indicating that there may be justification for indefinite treatment in some individuals.

Our patient had mild preexisting manifestations of psychiatric difficulties, as is commonly seen in the general population in childhood, including attentional challenges and anxiety. These were not disabling for this patient and she was functioning appropriately, performing well in academics and athletics. Additional challenging historical aspects included a positive family history of psychiatric conditions in a number of relatives, which suggested a familial etiology in this individual. Although a familial predisposition is often sought in psychiatric and neurologic conditions, caution should be exercised when attributing this to causation of symptoms in the proband [23]. There is the possibility that a propensity to an autoimmune substrate underlies psychiatric illness in some families, although this remains to be evaluated.

Our case emphasizes the need for a high index of suspicion for medical conditions in patients with new-onset psychiatric deterioration in childhood and adulthood. Confusing aspects, as in this case, may include a positive family history, or a relatively static or slowly deteriorating course, rather than a catastrophic presentation with acute psychosis progressing to a profound depression of level of consciousness. Inborn errors of metabolism can also present at any age with predominantly psychiatric manifestations and should be included in the differential diagnosis [24]. An important characteristic in any patient with psychiatric manifestations would be the cooccurrence of a movement phenomenon. Additionally, the unilateral nature of her manifestations raises concern regarding an underlying lesion that would necessitate, at the least, neuroimaging. This case also appears to confirm a propensity for unilateral symptoms in this condition as has been previously reported [5]. As noted, psychiatric conditions may be associated with comorbid movement disorders such as tic as in Tourette and stereotypy in autism. Conditions outside of these common comorbid movement disorders, such as ataxia, myoclonus, or Parkinsonism, should prompt further evaluation and referral to a neurologist. This case emphasizes the need for close coordination with neurology, psychiatry, and psychological services in situations when there is a possibility of an underlying identifiable neurologic substrate [25, 26].

## Supplementary Materials

Supplementary Materials Video demonstrating initial examination findings and changes during treatment course. At the time of initial evaluation, the patient demonstrates significant dysmetria on left sided finger to object testing. Gait demonstrates hypertonia of the ipsilateral leg with circumduction. As treatment is started she has improvements in the amplitude of the dysmetria, with more exact targeting. Symptoms persist however despite aggressive treatment.
